# Childhood Pars Planitis; Clinical Features and Outcomes

**Published:** 2011-10

**Authors:** Homayoon Nikkhah, Alireza Ramezani, Hamid Ahmadieh, Masoud Soheilian, Mohsen Azarmina, Mohammad-Hossein Dehghan, Siamak Moradian, Ramin Nourinia

**Affiliations:** Ophthalmic Research Center, Shahid Beheshti University of Medical Sciences, Tehran, Iran

**Keywords:** Childhood Uveitis, Pars Planitis, Visual Outcome, Complications

## Abstract

**Purpose:**

To evaluate the demographic and clinical features of childhood pars planitis, and to determine the therapeutic and visual outcomes of the disease.

**Methods:**

Medical records of pediatric patients (less than 16 years of age at diagnosis) with pars planitis and at least 6 months of follow-up who were referred to Labbafinejad Medical Center, Tehran, Iran over a 22 year period were reviewed.

**Results:**

Overall, 117 eyes of 61 patients including 51 (83.6%) male subjects were included. Mean age at the time of diagnosis was 7.8±3.2 (range, 3–16) years. Mean best corrected visual acuity (BCVA) was 0.88±0.76 logMAR at presentation which improved to 0.39±0.51 logMAR at final visit (P<0.001). Endotheliitis was present in 23 (19.6%) eyes and was significantly more prevalent in subjects younger than 9 years (P=0.025). Cataract formation (41.9%) and cystoid macular edema (19.7%) were the most prevalent complications. Univariate regression analysis showed that better baseline visual acuity (OR=0.38, 95%CI 0.21–0.70, P=0.002), age older than 5 years at disease onset (OR=0.36, 95%CI 0.14–0.9, P=0.029), absence of endotheliitis (OR=0.39, 95%CI 0.15–0.99, P=0.047) and female gender (OR=3.77, 95%CI 1.03–13.93, P=0.046) were significantly associated with final BCVA of 20/40 or better.

**Conclusion:**

Childhood pars planitis was much more common among male subjects. Endotheliitis may be a sign of inflammation spillover and is more prevalent in younger patients. Visual prognosis is favorable in most patients with appropriate treatment.

## INTRODUCTION

Pars planitis is a subset of intermediate uveitis characterized by aggregation of inflammatory cells and debris over the pars plana (snow banks) or in the vitreous cavity (snow balls) in the absence of infections or other known inflammatory conditions.^1^ It is a relatively common form of childhood and young adult-onset uveitis.^2^–^4^ The prevalence of pars planitis varies from 5% to 25% based on reports from different uveitis clinics.^5^–^7^

Considering the chronic, insidious nature of pars planitis and the fact that the anterior segment is often quiet in this condition, permanent visual loss may occur in children. There is lack of information on visual outcomes and complications of childhood pars planitis in Iran; we therefore reviewed medical records of patients with childhood pars planitis at our center spanning a time period exceeding 20 years.

## METHODS

Hospital records of all patients with childhood pars planitis who were referred to Labbafinejad Medical Center, a tertiary referral eye care center in Tehran-Iran from September 1983 to August 2005 were reviewed. Patients younger than 16 years with a classical presentation of pars planitis characterized by vitritis and presence of snow balls or snow banks, at least in one eye, were included. Patients with associated systemic or ocular disease, or abnormal laboratory tests compatible with a systemic condition known to be associated with intermediate uveitis were excluded. Only subjects with at least 6 months of follow-up were included for the purpose of the study.

Data included sex, age at onset of the disease, unilateral or bilateral involvement, initial ocular symptoms and signs, best-corrected visual acuity (BCVA) at presentation and at final examination based on Snellen chart or fixation pattern in preverbal/non-cooperative children, ocular complications and intraocular pressure (IOP). Ophthalmologic examination findings in detail included band keratopathy, endotheliitis, anterior chamber (AC) reaction, anterior vitreous reaction, anterior or posterior synechiae and cataracts on slit lamp biomicroscopy; and snow balls, snow banks, cyclitic membranes, peripheral retinal vasculitis, peripheral retinal neovascularization, papillitis or optic disc atrophy, and cystoid macular edema (CME) on fundus examination. CME was detected clinically or by fluorescein angiography. We also reviewed the results of laboratory tests and recorded treatment data including topical steroids, subtenon injections of corticosteroids, systemic steroids, immunosuppressive agents, laser photocoagulation and cryotherapy, as well as surgical procedures.

Statistical analysis was performed using SPSS software (Version 17.0, SPSS Inc., Chicago, USA). Wilcoxon signed rank test was used to evaluate the difference between initial and final BCVA. Regression analysis (univariate and multiple) was used to determine the effect of factors such as sex, age and BCVA at presentation on final BCVA.

## RESULTS

One hundred and seventeen eyes of 61 patients including 51 (83.6%) male and 10 (16.4%) female subjects were studied. Mean age at diagnosis was 7.8±3.2 (range, 3–16) years and mean follow-up duration was 44.6±37.8 (median, 34; range, 6–140) months. The condition was bilateral in 56 patients (91.8%). BCVA was based on fixation pattern in 7 eyes and 6 eyes had missing data. Mean BCVA was 0.88±0.76 (range, 0.12 to 2.9) logMAR at initial examination which was significantly improved to 0.39±0.5 (range, 0.12 to 2.6) logMAR at final follow-up; none of the BCVA data was based on fixation patterns at final examination. BCVA was 20/40 or better in 68 (58.1%) eyes and less than 20/200 in 13 (11.1%) eyes at final follow-up (Table 1).

The most common symptoms at presentation were blurred vision in 46 patients (75%), followed by red eye and eye deviation each in 6 subjects (10%). Floaters were the initial symptom in only 3 cases (5%). The most common ocular signs at presentation were snow banks in 82 (70.1%), snow balls in 67 (57.3%), vitritis in 63 (53.8%), periphlebitis in 44 (37.5%), anterior chamber reaction in 31 (26.5%), and endotheliitis in 23 (19.7%) eyes. The latter was significantly correlated with the age less than 9 years (P=0.025).

The most frequent complications were cataracts in 49 (41.9%), CME in 23 (19.7%), band keratopathy in 20 (17.1%), amblyopia in 20 (17.1%), epiretinal membranes (ERMs) in 9 (7.7%), ocular hypertension in 7 (6%), glaucoma in 7 (6%), vitreous hemorrhage in 6 (5.1%), retinal detachment in 5 (4.2%), and cyclitic membranes and optic atrophy each in 4 (3.4%) eyes. Posterior subcapsular cataracts were the most common form of lens opacities. CME and ERM were diagnosed clinically.

The therapeutic regimen included: topical corticosteroids in 40 eyes (34.2%), subtenon injection of triamcinolone in 86 eyes (73.5%) with a total of 253 injections (average of 3 injections per eye), oral prednisolone in 18 patients (29.5%), and oral cyclosporine (one capsule daily for 2 months) in one subject (0.9%). Peripheral retinal photocoagulation and cryotherapy were performed in 10 (8.5%) and 9 (7.7%) eyes respectively for treatment of peripheral retinal neovascularization. Cataract extraction was performed in 17 eyes including 11 eyes with severe lens opacity, of which 6 eyes underwent intraocular lens (IOL) implantation. Mean BCVA was not significantly different between IOL implanted (0.2 logMAR) and non-IOL implanted eyes (1.05 logMAR) (P=0.147). Six other eyes underwent lensectomy without IOL implantation as part of pars plana vitrectomy to facilitate vitreous base trimming. Pars plana vitrectomy was performed on 22 eyes due to complications of pars planitis. Indications for pars plana vitrectomy included severe vitreous organization (13 eyes), vitreous hemorrhage (4 eyes), rhegmatogenous retinal detachment (RRD, 2 eyes), ERM (2 eyes), tractional retinal detachment (1 eye), and previously failed scleral buckling (2 eyes). The retina was totally attached at final examination in all eyes; however 2 eyes required multiple vitreoretinal procedures.

During follow-up, IOP was higher than 21 mmHg in 14 eyes of 9 patients. IOP was controlled in 7 eyes of 5 patients by discontinuation of steroids; 4 eyes of 2 patients required antiglaucoma medications and 3 eyes of 2 patients underwent trabeculectomy.

Univariate logistic regression revealed that better BCVA at presentation (OR=0.38, 95%CI 0.21–0.70, P=0.002), age older than 5 years at disease onset (OR=0.36, 95%CI 0.14–0.9, P=0.029), female gender (OR=3.77, 95%CI 1.03–13.93, P=0.046) and absence of endotheliitis at initial examination (OR=0.39, 95%CI 0.15–0.99, P=0.047) were significantly correlated with final BCVA of 20/40 or better. However, multiple logistic regression disclosed that only BCVA at presentation (OR=0.37, 95%CI 0.18–0.74, P=0.004) and female gender (OR=10.75, 95%CI 1.21–95.8, P=0.033) were significantly correlated with final BCVA of 20/40 or better (Table 2).

## DISCUSSION

Pars planitis is considered a type of intermediate uveitis of unknown etiology associated with snow ball or snow bank formation. It is not associated with any systemic disease and is usually bilateral with asymmetric severity. Male to female ratio was 5 to 1 in our series, however the disease has been reported to be more frequent in female subjects in some other reports.^8^,^9^ The condition was bilateral in 92% of our patients. The rate of bilateral disease ranges from 84.3% as reported by Garcia et al^10^ down to 10% as described by Maris et al.^11^ Mean age at diagnosis was 7.8 years in our series which is close to corresponding figures of 9.2 years reported by Romero et al^12^, and 10 years by Arellanes-Garcia et al^10^.

Blurred vision was the most common symptom in our study which is consistent with other reports.^8^,^11^,^12^ Although patients may complain of decreased vision in one eye, both eyes are involved in most cases.^13^ Ocular deviation was the presenting symptom in 11 patients in our series which emphasizes the importance of a complete eye examination in any child with strabismus.

The most frequent signs in our study were snow banks (70.1%), snow balls (57.3%) and vitritis (53.8%). In a series by Raja et al^9^, vitritis (100%) and snow banking (91%) were the most common signs. Arellanes-Garcia et al^10^ reported vitritis (99.7%) and snow balls (99.3%) as the most frequent signs. Periphlebitis and anterior chamber reaction were other common signs on initial examination in our series, present in 37.5% and 26.5% of eyes respectively. Endotheliitis was noted in 23 eyes (19.7%) at presentation. Autoimmune endotheliopathy in pars planitis was first reported by Khodadoust et al^14^ in 4 of 10 patients. Corneal changes in this condition are similar to those seen in allograft corneal rejection. Although this finding may underscore the autoimmune nature of pars planitis, it has also been suggested to be due to spillover of inflammatory cells from the vitreous cavity.^15^

Cataract formation and CME have been reported as major complications of pars planitis.^8^,^10^,^12^ Cataracts (41.9%), CME (19.7%) and band keratopathy (17.1%) were the predominant complications in our series. Posterior subcapsular cataracts were the most common form of lens opacity. Romero et al^12^ reported cataracts (36.7%), ocular hypertension (23.3%) and retinal neovascularization (23.3%) as the most frequent complications of pars planitis in their series. Cataracts (30.4%), CME (26.1%) and ERM (26%) were reported as the most prevalent complications by Donaldson et al.^8^ In a Mexican Mestizo population, Arellanes-Garcia et al^10^ reported CME (63.4%) and cataracts (47.5%) to be the most common complications. Cataracts may develop due to corticosteroid therapy or as a sequel to ocular inflammation.

In the current study, cataract surgery was performed in 11 eyes because of severe lens opacity and in 6 eyes as part of pars plana deep vitrectomy procedures. Six eyes in the first group underwent IOL implantation whereas in the second group, all 6 eyes were left aphakic. Tessler et al^16^ randomized 26 patients with chronic iridocyclitis or pars planitis to cataract surgery with or without IOL implantation and found no statistically significant difference in visual outcomes between the two groups, a finding in line with our results which revealed IOL implantation is a safe procedure with good results (mean BCVA of 0.2 logMAR).

Fourteen eyes (12%) in our series developed ocular hypertension during follow-up, of which 7 (6%) responded favorably to steroid discontinuation. The remaining 7 eyes (6%) developed glaucoma; in 4 eyes, the glaucoma was controlled with medications, but 3 other eyes underwent trabeculectomy. In the series reported by Romero et al^12^, ocular hypertension occurred in 23.3% of eyes but none of them developed glaucoma. Arellanes-Garcia^10^ reported ocular hypertension and glaucoma in 22.7% and 7.5% of eyes in their series, respectively. Filtering surgery was required in half of the patients with glaucoma.

Vitreous hemorrhage occurred in 5 eyes (4.2%) which resolved spontaneously in one eye; the other four eyes underwent pars plana deep vitrectomy due to nonclearing vitreous hemorrhage. Mean BCVA in these eyes was 0.19 logMAR. Visual outcomes of pars plana vitrectomy for vitreous hemorrhage, as reported by Potter et al,^17^ are generally good. Vitreous hemorrhage is not a frequent complication of pars planitis.^11^,^12^,^18^

In the current study, retinal detachment (RD) occurred in 5 eyes of 5 patients (4.2%) including tractional RD in one eye and RRD in 4 eyes. Romero and coworkers^12^ reported 3 patients with RD (10%) in their study. Arellanes-Garcia et al^10^ reported RRD in 5 patients (1.7%). In the series reported by Malinowski et al^19^, RD occurred in 9 eyes (8.3%). In our series 3 patients underwent pars plana deep vitrectomy and 2 others underwent scleral buckling for RD. In two eyes retinal reattachment was achieved after multiple vitreoretinal procedures. Mean BCVA in RD patients was 0.76 logMAR postoperatively. Thirteen eyes of 11 patients underwent pars plana deep vitrectomy for dense vitreous organization and achieved mean final BCVA of 0.42 logMAR.

Treatment of pars planitis needs a stepwise and individualized approach. In our series, 22 eyes with mild vitreous involvement, good visual acuity (>20/40) and no vision threatening complications, received no treatment. In eyes with moderate to severe vitritis, periocular steroid injections (mostly subtenon) were the mainstay of therapy. Oral corticosteroids were given in subjects with poor response to periocular injections or in cases with severe bilateral disease. Topical corticosteroids were added if there was associated anterior chamber reaction. Systemic immunosuppressive agents were required only in one patient to control the inflammation. Cryotherapy and laser photocoagulation were the last options, as suggested by Whitcup.^20^ Complications intractable to medical therapy were managed by surgical means.

In our series, mean BCVA at presentation was 0.88 logMAR which was improved to 0.39 logMAR at final examination (P<0.001). BCVA of 20/40 or better was present in 37% of eyes at presentation and in 68% of eyes at final follow-up; 40% of eyes at presentation and 13% at final follow-up had BCVA less than 20/200. Romero et al^12^ reported mean BCVA of 0.64 and 0.84 logMAR at initial and final examinations, respectively. Corresponding values were 0.36 and 0.34 logMAR in the study by Malinowski et al^19^. Both univariate and multivariate regression analyses showed that final BCVA of 20/40 or better was significantly correlated with better BCVA at presentation and with female gender. Better baseline BCVA indicates milder disease and low grade ocular involvement, on the other hand it may reflect earlier diagnosis and therefore forecasts better visual outcomes. The authors have no explanation for the fact that female gender was a good prognostic factor. Absence of endotheliitis at presentation and age above 5 years at onset were significantly associated with final BCVA of 20/40 or better only based on univariate analysis. Since pars planitis is often quiet and insidious, young children and their parents fail to notify any problem in the early stages of the disease leading to delayed diagnosis and poor visual outcomes.

The major limitation of our study is its retrospective nature; some data such as BCVA at presentation in 6 eyes and final BCVA in 6 other cases were missing. Moreover, the study was performed at a tertiary referral center and therefore mild cases were probably not included. Therefore these results may not be generalizable to all pediatric patients with pars planitis. The major conclusion of this study is that, childhood pars planitis is a treatable disease with good visual prognosis as long as early diagnosis, appropriate treatment and regular follow-up are provided.

## Figures and Tables

**Table 1:** Best-corrected visual acuity (BCVA) at initial and final examinations

BCVA	Number (percent)	
	Initial	Final
≥ 20/40	37 (31.6)	68 (58.1)
20/50–20/200	25 (21.4)	30 (25.6)
<20/200	42 (35.8)	13 (11.1)
Missing data	6 (5.2)	6 (5.2)
CSM	7 (6.0)	0 (0)
M±SD (logMAR)	0.88±0.76	0.39±0.51*

* P<0.001, Wilcoxon Signed-Rank test.

CSM, central, steady, maintained; M, mean; SD, standard deviation

**Table 2:** Effect of different factors on final best corrected visual acuity (BCVA) of 20/40 or better

	Univariate analysis*		Simultaneous effect**	
	OR (95% CI)	P	OR (95% CI)	P
BCVA at presentation	0.38 (0.21 – 0.70)	0.002	0.37 (0.18 – 0.74)	0.004
Age ≤5 years at presentation	0.36 (0.14 – 0.90)	0.029	0.55 (0.15 – 2.03)	0.413
Phlebitis	1.26 (0.48 – 3.27)	0.642	1.26 (0.3 – 5.17)	0.834
AC reaction	0.64 (0.27 – 1.48)	0.293	0.61 (0.2 – 1.93)	0.366
Endotheliitis	0.39 (0.15 – 0.99)	0.047	0.5 (0.15 – 1.7)	0.408
Sex (female)	3.77 (1.03 – 13.93)	0.046	10.75 (1.21 – 95.8)	0.033

* Based on logistic regression;

** Based on multiple logistic regression

OR, odds ratio; CI, confidence interval; AC, anterior chamber
